# Clinical Severity of Diabetic Ketoacidosis by SGLT2 Inhibitors Use: A Retrospective Observational Study

**DOI:** 10.1002/dmrr.70226

**Published:** 2026-09-07

**Authors:** Mor Naaman, Tamar Fisher‐Negev, Genya Aharon‐Hananel, Tehila Harel, Avivit Cahn

**Affiliations:** ^1^ The Faculty of Medicine Hebrew University of Jerusalem Jerusalem Israel; ^2^ Department of Orthopedics Hadassah Medical Center Jerusalem Israel; ^3^ School of Pharmacology Faculty of Medicine Hebrew University of Jerusalem Jerusalem Israel; ^4^ Diabetes Unit Department of Endocrinology and Metabolism Hadassah Hebrew University Hospital Jerusalem Israel; ^5^ Internal Medicine Department A Hadassah Hebrew University Hospital Jerusalem Israel

**Keywords:** diabetic ketoacidosis, euglycemic diabetic ketoacidosis, sodium‐glucose cotransporter 2 inhibitors, type 1 diabetes, type 2 diabetes

## Abstract

**Aims:**

Sodium glucose cotransporter 2 inhibitors (SGLT2i) are commonly used glucose lowering agents with established cardiac and kidney benefits. Diabetic ketoacidosis (DKA) is a rare complication associated with their use, which entails significant morbidity and mortality.

**Materials and Methods:**

This retrospective observational study included patients with diabetes (any) admitted with DKA to a large tertiary hospital. Demographic, clinical and laboratory data were extracted from electronic medical records. Outcomes included DKA severity as determined by nadir pH, duration of stay, need for intensive care (ICU) and inpatient mortality.

**Results:**

Baseline SGLT2i use was documented in 27/191 patients (14.1%). SGLT2i users had lower nadir pH (7.08 ± 0.15 vs. 7.15 ± 0.12, *p* = 0.006) and were more likely to have severe DKA (29.6% vs. 12.2%, *p* = 0.007). After adjusting for multiple covariates, SGLT2i use remained independently associated with DKA severity. ICU admission and hospitalisation duration did not differ between the groups. Inpatient mortality occurred in 3/27 users and 3/164 non‐users, while the composite outcome of mortality and/or ICU admission was similar (74.1% vs. 72.0%, *p* = 0.819). Findings remained consistent after excluding patients with new‐onset diabetes.

**Conclusions:**

Among patients hospitalised with DKA in a large tertiary centre, SGLT2i use was associated with a more severe biochemical presentation, reflected by a lower nadir pH. The observed difference in inpatient mortality was based on few events and should be interpreted cautiously.

## 1 Introduction

Sodium‐glucose cotransporter 2 inhibitors (SGLT2i) are glucose‐lowering agents used primarily for the treatment of type 2 diabetes (T2D) [1]. They have demonstrated cardiovascular and renal benefits in patients with and without diabetes [2, 3, 4, 5, 6, 7, 8].

SGLT2i use is associated with an approximately twofold increased risk of diabetic ketoacidosis (DKA) [9, 10, 11, 12]. The proposed mechanism is multifactorial: SGLT2i‐induced glycosuria lowers insulin availability and increases the glucagon‐to‐insulin ratio, thereby promoting lipolysis and hepatic ketogenesis [13]. SGLT2i‐associated DKA may present with marked hyperglycaemia or as euglycaemic DKA, characterised by only mildly elevated or normal glucose levels [13].

SGLT2i have been evaluated as adjunctive therapy in patients with type 1 diabetes (T1D) because they may improve glycaemic control, promote weight loss and reduce glycaemic variability [14, 15, 16]. However, their use may increase DKA risk up to six fold in patients with T1D [17]. Although European regulators previously approved dapagliflozin for selected patients with T1D, this indication was later withdrawn [14, 15]. Nevertheless, some clinicians continue to prescribe SGLT2i off label in some settings, including in Israel [18].

Only limited evidence has examined whether baseline SGLT2i use affects DKA severity [19]. We aimed to determine whether DKA severity, assessed by nadir venous pH, and clinical outcomes differed between baseline SGLT2i users and non‐users.

## 2 Materials and Methods

### 2.1 Patients and Setting

This retrospective observational study utilised anonymised data of patients with diabetes, with and without baseline use of SGLT2 inhibitors admitted to both campuses of the Hadassah Medical Center with a diagnosis of DKA between December 1, 2018, and December 31, 2023.

Adult patients admitted to the hospital with a diagnosis of DKA were included in the analysis. Exclusion criteria included: (1) Lowest pH during admission > 3; (2) seemingly alternative causes for acidosis; (3) Lactate level > 3 ULN (upper limit of normal); (4) non‐diabetic patients; (5) pregnant women; and (6) dialysis. In the case of multiple episodes of DKA in the same patient, only the first episode was considered.

All data, including demographics, medical diagnoses, chronic medications, laboratory test results and hospitalizations, were captured from the Hadassah computerised database.

The study was conducted in accordance with the Declaration of Helsinki and was approved by the Institutional Review Board (IRB) of Hadassah Medical Center. The requirement for informed consent was waived due to the retrospective nature of the study.

### 2.2 Definition of Variables

DKA cases were identified using the following ICD‐9 diagnostic codes: 250.11–250.13 for diabetic ketoacidosis, and 276.2 for metabolic acidosis, in combination with a diabetes diagnosis coded as 250.00–250.03. DKA was defined according to The American Diabetes Association criteria, including (1) pH ≤ 7.3; (2) metabolic acidosis with a positive anion gap; (3) presence of ketones in blood or urine; and (4) elevated glucose levels. Because euglycaemic DKA is a recognized presentation, cases fulfiling DKA criteria despite the absence of overt hyperglycaemia were also included when a prior history of diabetes was documented [20, 21].

Baseline use of the SGLT2 inhibitor was captured according to the list of drugs on admission or based upon the free text listed in the admission chart. Severity of DKA was assessed based on the lowest pH value measured during hospitalisation, according to the definition of The American Diabetes Association [20]: mild DKA was defined as pH between 7.25 and 7.30, moderate DKA as pH between 7.00 and 7.24, and severe DKA as pH below 7.00.

The lowest creatinine measurement during admission was considered as reflective of the baseline, chronic renal status. Baseline levels were not reflective of the chronic kidney state because of the high frequency of acute kidney injury in this population. eGFR was calculated according to age, sex, and SCr, using the 2021 CKD‐EPI equation, and level of kidney function was categorised as: [1] eGFR ≥ 60 mL/min/1.73 m^2^ [2] eGFR < 60 mL/min/1.73 m^2^ [22].

Symptoms and triggers of the DKA event were collected from the free text in the admission chart and grouped into categories by the clinical judgement of the first author and discussed with the senior author in case of uncertainty.

### 2.3 Outcomes

Outcomes were assessed by baseline use of SGLT2 inhibitors. The primary endpoint was the severity of the DKA episode as measured by the lowest pH during admission (as the nadir pH may have occurred several hours post admission); pH was considered both as a continuous and as a categorical variable.

Secondary endpoints included duration of hospitalisation, ICU admission and its duration, level of consciousness, in‐patient mortality, and the combination of in‐patient mortality and ICU admission.

### 2.4 Statistical Analyses

Baseline characteristics are reported as frequencies and percentages for categorical variables, and as mean and standard deviation (SD) or median and interquartile range (IQR) for continuous variables. Comparisons between groups (SGLT2 inhibitors users vs. non‐users) were performed using Student's *t*‐test or Mann‐Whitney *U* test for continuous variables, and Chi‐square test or Fisher's exact test for categorical variables, as appropriate.

To further evaluate the association between the severity of DKA and SGLT2 inhibitor use, a stepwise ANCOVA model was constructed using nadir pH as the continuous dependent variable. Variables included in the initial model were selected based on statistical significance in preliminary univariate *t*‐tests, and included: age, sex, eGFR, relevant comorbidities, and SGLT2 inhibitor use. A stepwise selection method was used to identify the most relevant predictors. A sensitivity analysis using a categorical regression model based on DKA severity classes was also used to examine the association between SGLT2 inhibitor use and severity of the DKA.

A sensitivity analysis excluded all patients in whom the DKA episode was the clinical presentation of new onset T1DM. All had not been using SGLT2 inihibitors at baseline.

Additional subgroup analyses were performed separately for patients with type 1 and type 2 diabetes.

No formal adjustment for multiple comparisons was applied. Nadir pH analysed as a continuous variable was the primary endpoint, whereas secondary, sensitivity, and subgroup analyses were considered exploratory; nominal two‐sided *p*‐values are therefore reported.

A two‐tailed *p*‐value < 0.05 was considered statistically significant. Analyses were conducted using SPSS version 30.0 (IBM Corp.).

## 3 Results

### 3.1 Study Cohort

**FIGURE 1:**
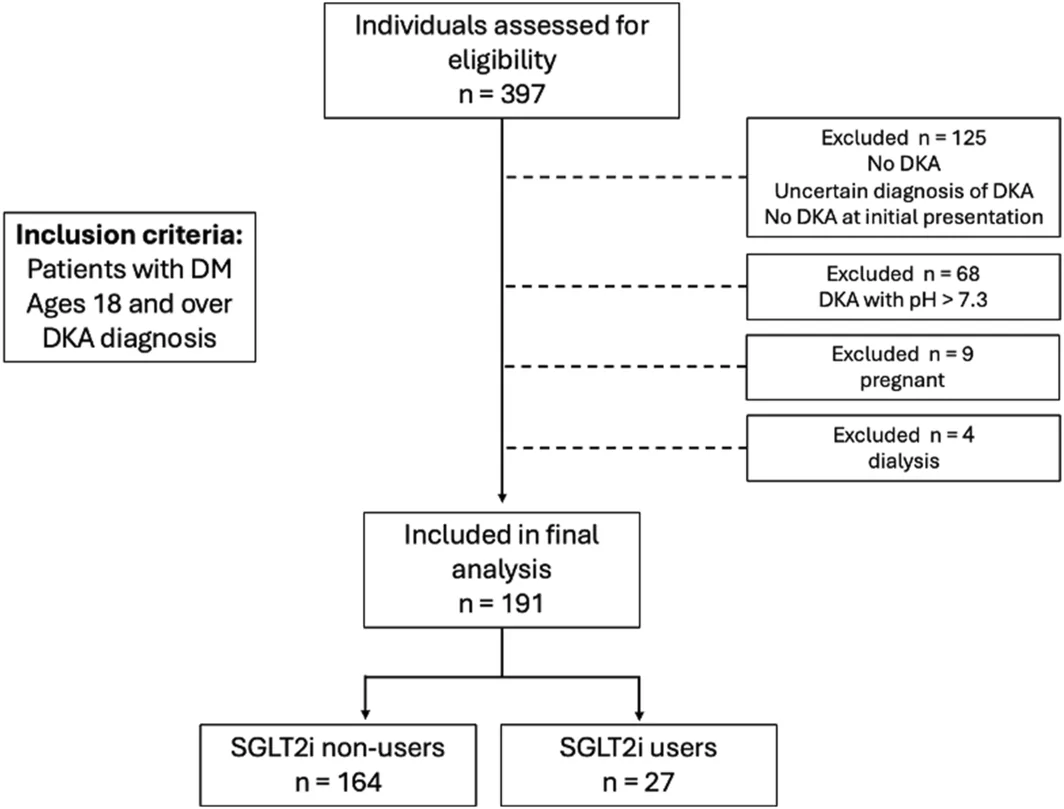
Study flow diagram. DKA, diabetic ketoacidosis; DM, diabetes mellitus; SGLT2i, sodium‐glucose co‐transporter 2 inhibitors.

A total of 397 patients with the relevant diagnostic codes were extracted from the medical files and assessed for eligibility (Figure 1). After applying the exclusion criteria, 191 patients were included in the final analyses; their baseline characteristics are shown in Table 1. Among these, 27 patients were identified as baseline users of SGLT2 inhibitors, and 164 patients were non‐users.

**TABLE 1:** Baseline characteristics.

	Overall *n* = 191	SGLT2i users *n* = 27	SGLT2i non‐users *n* = 164	*p*‐value
Age, years	42.6 ± 20.6	65.5 ± 15.7	38.9 ± 18.8	0.0001
Sex				
Female	103 (53.9%)	10 (37.0%)	93 (56.7%)	0.057
Male	88 (46.1%)	17 (63.0%)	71 (43.3%)	
DM type				
Type 1	132 (69.1%)	6 (22.2%)	126 (76.8%)	0.0001
Type 2	53 (27.7%)	20 (74.1%)	33 (20.1%)	
Other^a^	6 (3.1%)	1 (3.7%)	5 (3.0%)	
New onset of DM	31 (16.2%)	0 (0.0%)	31 (18.9%)	0.009
Baseline use of insulin	131 (68.6%)	13 (48.1%)	118 (72.0%)	0.014
Congestive heart failure (CHF)	6 (3.1%)	4 (14.8%)	2 (1.2%)	0.004
Ischaemic heart disease	16 (8.4%)	7 (25.9%)	9 (5.5%)	0.002

*Note:* Data are presented as mean ± SD or *n* (%), as appropriate.

Abbreviations: CHF, congestive heart failure; DM, diabetes mellitus; SD, standard deviation; SGLT2i, sodium‐glucose cotransporter 2 inhibitor.

^a^ Other—maturity‐onset diabetes of the young, pancreatitis, other.

### 3.2 Baseline Characteristics

SGLT2 inhibitor users were significantly older than non‐users (65.5 ± 15.7 vs. 38.9 ± 18.8 years, *p* = 0.0001). Most of the patients in our study had T1DM (69.1%), yet most SGLT2 inhibitor users had T2D (74.1%). Among the 27 SGLT2i users, 6 had T1DM, 20 had T2DM, and one had another type of diabetes. In 16.2% of the overall patients, the DKA episode was the initial presentation of T1D, and none of them had been using SGLT2 inhibitors at baseline. Regarding baseline comorbidities, congestive heart failure (CHF) was significantly more prevalent among SGLT2 inhibitor users compared with non‐users (14.8% vs. 1.2%, *p* = 0.004). Similarly, ischaemic heart disease was more common in the users' group (25.9% vs. 5.5%, *p* = 0.002).

### 3.3 Precipitating Events and Symptoms

Precipitating events and presenting symptoms are summarised in Supporting Information S1: Table S1. Overall, signs and symptoms were similar between SGLT2 inhibitor users and non‐users. Nausea or vomiting (68.6%) and weakness or fatigue (61.8%) were the most common presenting symptoms and did not significantly differ by SGLT2 inhibitor use. Thirst or frequent urination was less common among SGLT2 inhibitor users (7.4% vs. 26.8%, *p* = 0.03). Infectious illness was the most common precipitating factor (35.6%). Reduced insulin was reported less frequently among SGLT2 inhibitor users (22.2% vs. 48.8%, *p* = 0.01). Most patients were alert at presentation (83.2%), with no difference between groups.

### 3.4 Laboratory Tests

**FIGURE 2:**
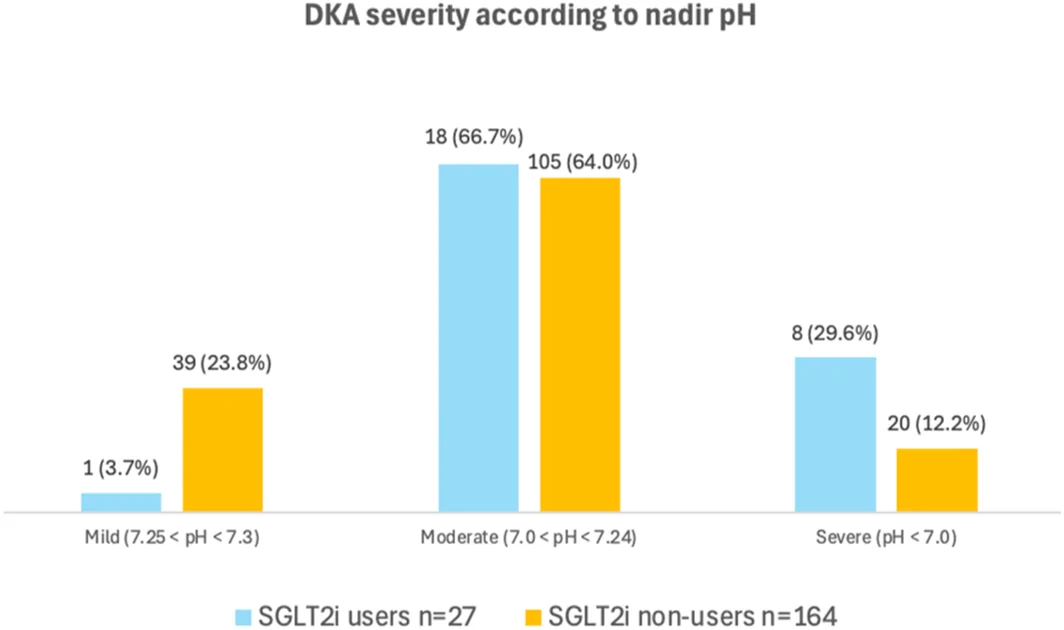
DKA severity according to nadir pH among SGLT2i users and non‐users. Values are presented as *n* (%). DKA, diabetic ketoacidosis; SGLT2i, sodium‐glucose cotransporter 2 inhibitors.

As shown in Table 2, SGLT2i users exhibited lower nadir pH values compared to non‐users (mean pH 7.08 ± 0.15 vs. 7.15 ± 0.12, *p* = 0.006). DKA severity, classified by pH categories, also differed significantly between groups (*p* = 0.007); 66.7% of SGLT2i users had moderate DKA and 29.6% had severe DKA, whereas among non‐users, 64% were moderate and only 12.2% were severe (Figure 2). Initial pH values tended to be lower in SGLT2i users (7.11 vs. 7.16), but this difference did not reach statistical significance (*p* = 0.08). Euglycaemic DKA (glucose < 250 mg/dL) was more common among SGLT2 inhibitor users compared with non‐users (18.5% vs. 5.5%, *p* = 0.031). Baseline kidney function (as approximated based on the lowest creatinine during admission) differed significantly between the groups. Among SGLT2i users, 22.2% had eGFR < 60 ml/min/1.73 m^2^ compared with 5.5% among non‐users (*p* = 0.003).

**TABLE 2:** Laboratory values.

	Overall *n* = 191	SGLT2i users *n* = 27	SGLT2i non‐users *n* = 164	*p*‐value
Labs at baseline				
pH	7.15 ± 0.13	7.11 ± 0.17	7.16 ± 0.12	0.080
Glucose (mg/dL)	494.73 ± 184.97	397.6 ± 171.5	510.7 ± 182.7	0.002
Euglycaemic DKA (glucose < 250 mg/dL)	14 (7.3%)	5 (18.5%)	9 (5.5%)	0.031
CO2 levels (mmol/L)	26.8 ± 7.83	26.7 ± 8.9	26.8 ± 7.6	0.866
Anion gap	25.6 ± 7.0	28.3 ± 8.4	25.14 ± 6.6	0.030
Creatinine (at time of admission), mg/dL	1.52 ± 0.84	1.73 ± 0.71	1.49 ± 0.85	0.088
Labs during hospitalisation				
Nadir pH	7.14 ± 0.13	7.08 ± 0.15	7.15 ± 0.12	0.006
DKA severity by nadir pH levels				
Mild (7.25 < pH < 7.3)	40 (20.9%)	1 (3.7%)	39 (23.8%)	0.007
Moderate (7.0 < pH < 7.24)	123 (64.4%)	18 (66.7%)	105 (64.0%)	
Severe (pH < 7.0)	28 (14.7%)	8 (29.6%)	20 (12.2%)	
Lowest creatinine, mg/dL	0.71 ± 0.45	0.85 ± 0.51	0.68 ± 0.44	0.231
eGFR (ml/min/1.73 m^2^) based on lowest creatinine	112.5 ± 32.2	91.37 ± 32.7	118.24 ± 27.33	< 0.001
eGFR categories (baseline), ml/min/1.73 m^2^				
eGFR ≥ 60	176 (92.15%)	21 (77.8%)	155 (94.5%)	0.003
eGFR < 60	15 (7.85%)	6 (22.2%)	9 (5.5%)	

*Note:* Data are presented as mean ± SD or *n* (%), as appropriate.

Abbreviations: CO_2_, carbon dioxide; DKA, diabetic ketoacidosis; eGFR, estimated glomerular filtration rate; SD, standard deviation; SGLT2i, sodium‐glucose cotransporter 2 inhibitor.

### 3.5 Clinical Outcomes

Inpatient mortality occurred in 3 of 27 SGLT2i users (11.1%) and 3 of 164 non‐users (1.8%, *p* = 0.038). However, the composite outcome of inpatient mortality and/or ICU admission did not differ between groups (74.1% vs. 72.0%, *p* = 0.819). ICU admission rates, duration of ICU stay, and total hospitalisation duration were also similar between groups (Table 3).

**TABLE 3:** Clinical outcomes.

	Overall *n* = 191	SGLT2i users *n* = 27	SGLT2i non‐users *n* = 164	*p*‐value
Inpatient mortality	6 (3.1%)	3 (11.1%)	3 (1.8%)	0.038
ICU hospitalisation	134 (70.2%)	17 (63.0%)	117 (71.3%)	0.378
Mean time in ICU, days	2.03 ± 3	1.52 ± 1.53	2.12 ± 3.2	0.440
Mortality and/or ICU	138 (72.3%)	20 (74.1%)	118 (72.0%)	0.819
Duration of hospitalisation, days	5.9 ± 6.3	6.9 ± 7.4	5.7 ± 6.0	0.482

*Note:* Data are presented as mean ± SD or *n* (%), as appropriate.

Abbreviations: ICU, intensive care unit; SD, standard deviation; SGLT2i, sodium‐glucose cotransporter 2 inhibitor.

### 3.6 Independent Predictors of DKA Severity

In multivariate models, the severity of the DKA episode, as reflected by nadir pH, was independently associated with the use of SGLT2 inhibitors (Adjusted OR = 5.48, 95% CI 1.81–16.64, *p* = 0.003 for categorical pH, and *p* = 0.041 for continuous pH; Supporting Information S1: Table S2).

### 3.7 Sensitivity Analyses

In a sensitivity analysis, we excluded all patients with new‐onset diabetes (*n* = 31) and a total of 160 patients remained. The baseline characteristics of this restricted cohort were nearly identical to those of the full study population (Supporting Information S1: Table S3). Symptoms, triggers and clinical outcomes maintained similar patterns. Findings related to pH remained robust and statistically significant, including lower nadir pH levels and a higher proportion of severe DKA among SGLT2 inhibitor users. Predictors of DKA were similar as well (Supporting Information S1: Table S4).

### 3.8 Subgroup Analysis by Diabetes Type

In the T2DM subgroup, 20 patients were SGLT2i users and 33 were non‐users. SGLT2i users had a lower nadir pH (7.07 ± 0.16 vs. 7.15 ± 0.10, *p* = 0.036), and severe DKA occurred in 6 of 20 users (30.0%) compared with 1 of 33 non‐users (3.0%, *p* = 0.013). In the T1DM subgroup, only 6 patients were SGLT2i users, compared with 126 non‐users. Nadir pH did not differ significantly between groups (7.08 ± 0.14 vs. 7.14 ± 0.12, *p* = 0.239), and severe DKA occurred in 2 of 6 users (33.3%) and 19 of 126 non‐users (15.1%, *p* = 0.243). Other baseline characteristics, laboratory values, and clinical outcomes showed no consistent significant differences between SGLT2i users and non‐users in the stratified analyses, although age remained significantly higher among SGLT2i users in both subgroups (Supporting Information S1: Tables S5 and S6). Given the modest subgroup sizes, particularly the six SGLT2i users with T1DM, these findings should be considered exploratory.

## 4 Discussion

In this retrospective observational study, SGLT2i users hospitalised with DKA had a more severe biochemical presentation, reflected by lower nadir pH, compared with non‐users. Inpatient mortality was proportionally higher among SGLT2i users; however, this finding was based on only three deaths in each group and should be interpreted cautiously. In multivariable analysis, SGLT2i use remained independently associated with lower pH, alongside CHF and CKD. Findings regarding DKA severity remained consistent after excluding patients with new‐onset diabetes.

SGLT2i users also had a substantially higher prevalence of cardiovascular comorbidities, including congestive heart failure and ischaemic heart disease. This imbalance may partly reflect preferential prescribing of SGLT2i to patients with established cardiorenal disease and may identify a clinically older and higher‐risk population with potentially reduced physiological reserve. These baseline differences may therefore have contributed to the observed differences in DKA severity and clinical outcomes. Congestive heart failure remained independently associated with nadir pH in the multivariable model, whereas ischaemic heart disease was not retained. Nevertheless, residual confounding by indication cannot be excluded, and the observed association should not be attributed solely to SGLT2i exposure.

Several mechanisms may explain the severe presentation of DKA in patients taking SGLT2 inhibitors. By promoting urinary glucose excretion, SGLT2 inhibitors lower circulating glucose levels, even during significant insulin deficiency. This can lead to substantial ketoacidosis without profound hyperglycaemia. The absence of marked hyperglycaemia may delay the recognition of DKA by both patients and clinicians, allowing the acidosis to progress unnoticed and resulting in more advanced acidemia at the time of presentation. Moreover, SGLT2 inhibitors mimic fasting state shifting metabolism toward enhanced lipolysis and ketogenesis. They reduce insulin levels and increase glucagon concentrations, creating a hormonal environment that favours accelerated hepatic ketone production. This metabolic shift can deepen the degree of ketoacidosis once it begins, thereby lowering pH more profoundly than in patients not taking these agents.

A retrospective multicentre study from Qatar evaluated predictors of DKA severity and found that lower pH was associated with prolonged hospitalisation. In our cohort, hospitalisation duration did not differ between SGLT2 inhibitor users and non‐users [23]. Nevertheless, the lower nadir pH observed among SGLT2i users suggests a more severe biochemical presentation, even though this did not translate into longer hospital stays. Inpatient mortality was proportionally higher among SGLT2i users; however, this finding was based on only three deaths in each group, and the composite outcome of inpatient mortality and/or ICU admission did not differ between groups.

A recent multicentre UK study by Sharma et al. similarly found lower admission glucose and more pronounced acidosis among SGLT2i users with T2D, while ICU admission, length of stay, and mortality were comparable between users and non‐users [24]. In addition, a recent pharmacovigilance study of older adults with SGLT2i‐associated DKA found that lower admission pH and infection were associated with ICU admission [25], suggesting that severe acidosis may identify higher‐risk presentations in older patients.

However, other recent studies have reported less consistent associations between SGLT2i use and DKA severity or clinical outcomes. Another large Qatari cohort identified no significant differences in biochemical severity or clinical outcomes and attributed only a minority of DKA cases directly to SGLT2 inhibitor use [26]. In the Qatari cohort reported by Eledrisi et al., SGLT2i use was low (6.4% of patients with T2D and 0.17% of patients with T1D), particularly among patients with T1D, which likely contributed to the limited observed association between SGLT2i exposure and DKA severity.

Similarly, a study from Shamir Medical Centre regarding the severity of DKA among T2D patients found no difference in DKA severity between SGLT2 inhibitor users and non‐users, despite similar trends of lower glucose levels among users [27]. This disparity may be explained by methodological and population‐related distinctions. Although their analysis included a comparison between SGLT2 inhibitor users and non‐users, this was not the primary focus of the study, which was centred mainly on differences between T1D and T2D. Additionally, all 25 patients treated with SGLT2 inhibitors had T2D, whereas our cohort included both T2D patients and individuals with T1D treated off label. These clinical differences may influence DKA physiology and severity.

An additional Israeli study by Rotman‐Pikielny et al. [28] found that DKA hospitalisation rates remained stable following the introduction of SGLT2i. Only seven SGLT2i‐related DKA cases were identified, and their clinical characteristics were similar to those of the remaining cohort.

A notable strength of this study is its use of real‐world clinical data from two hospital campuses, which is likely to reduce selection bias and improve the generalisability of the findings within our healthcare system. In addition, the study included a relatively large cohort of confirmed DKA cases compared with previous single‐centre reports, as well as the use of nadir pH—a robust and objective marker of biochemical severity. The consistency of the results across multiple multivariable models further enhances the internal validity of our conclusions.

However, several limitations should be acknowledged. As with any retrospective observational study, causality cannot be inferred, and the findings should be interpreted as associations rather than evidence of a causal relationship. Furthermore, HbA1c levels were not available, as access to outpatient medical records is restricted due to ethical and data governance limitations. This precluded the assessment of long‐term glycaemic control and its potential contribution to DKA severity. The statistical modelling approach relied on the stepwise selection of variables identified in univariate analyses. Given the relatively small sample size, the potential for model overfitting should be acknowledged, and the stability of the identified predictors should be interpreted with caution. In addition, no formal adjustment was made for multiple comparisons; therefore, findings from secondary, sensitivity, and subgroup analyses should be interpreted as exploratory. Moreover, mortality comparisons were based on a very small number of events, particularly within the SGLT2 inhibitor group, and should therefore be interpreted cautiously and considered exploratory. Finally, the number of patients treated with SGLT2 inhibitors remained modest, which limits statistical power, particularly for subgroup analyses, and increases uncertainty around effect estimates. In addition, the proportion of SGLT2 inhibitor users in our cohort was relatively high compared to prior studies, which may reflect local prescribing patterns, referral bias, and inclusion of a higher‐risk population, and may limit the generalisability of the findings.

In conclusion, our study found a more severe presentation of DKA in SGLT2 inhibitor users versus nonusers. Prescribers are advised to exercise caution and ensure patients are well‐informed about ‘sick day rules,’ including the need to temporarily discontinue SGLT2 inhibitors and adjust insulin dosing as appropriate.

## Author Contributions

**Mor Naaman:** conceptualization, methodology, investigation, data curation, visualization, writing – original draft, writing – review and editing. **Tamar Fisher‐Negev:** supervision, interpretation of data, writing – review and editing. **Genya Aharon‐Hananel:** supervision, writing – review and editing. **Tehila Harel:** supervision, writing – review and editing. **Avivit Cahn:** conceptualization, methodology, supervision, interpretation of data, validation, writing – review and editing. All authors reviewed and approved the final version of the manuscript.

## Funding

The authors have nothing to report.

## Conflicts of Interest

G.A.‐H. reports travel support from Novo Nordisk and Medetronic through Hadassah Medical Center and AstraZeneca through Sheba medical centre. Speaker's Bureau: AstraZeneca, Novo Nordisk, Eli Lilly and Sanofi. A.C. has received advisory board and/or honoraria from Novonordisk, Sanofi, Boehringher Ingelheim, AstraZeneca, Elli Lilly, Rafa, Bayer and MedialEarlySign. M.N., T.F.‐N. and T.H. have no conflicts to declare.

## Supporting information


Supporting Information S1

## Data Availability

The data that support the findings of this study are available on request from the corresponding author. The data are not publicly available due to privacy or ethical restrictions.
