# Supplementary material for: Clinical Severity of Diabetic Ketoacidosis by SGLT2 Inhibitors Use: A Retrospective Observational Study
Source: Diabetes Metab Res Rev. 2026 Sep 7;42(6):e70226. doi: 10.1002/dmrr.70226 (PMC13549235; doi:10.1002/dmrr.70226)
Supplement: Supplementary file 1 [file DMRR-42-e70226-s001.docx]

Table of Contents- Supplements

[Table S1- Overall population, Symptoms and Triggers	2](#_Toc238127132)

[Table S2- Multivariate analysis of predictors of DKA severity for the overall population	4](#_Toc238127133)

[Table S3- Sensitivity analyses excluding patients with new onset of diabetes	5](#_Toc238127134)

[Table S4- Multivariate analysis of predictors of DKA severity excluding patients with new diabetes	7](#_Toc238127135)

[Table S5- Subgroup analysis of T1DM patients	8](#_Toc238127136)

[Table S6- Subgroup analysis of T2DM patients	10](#_Toc238127137)

# ***Supplements***

## **Table S1- O*verall population, Symptoms and Triggers***

|  | **Overall n = 191** | **SGLT2i users n = 27** | **SGLT2i non-users n = 164** | **p-value** |
| --- | --- | --- | --- | --- |
| ***Signs and Symptoms*** | | | | |
| Nausea or vomiting | 131 (68.6%) | 17 (63.0%) | 114 (69.5%) | 0.508 |
| Abdominal pain | 84 (44.0%) | 10 (37.0%) | 74 (45.1%) | 0.433 |
| Fever | 10 (5.2%) | 1 (3.7%) | 9 (5.5%) | 1.0 |
| Shortness of breath | 86 (45.0%) | 16 (59.3%) | 70 (42.7%) | 0.109 |
| Fruited scented breath | 12 (6.3%) | 1 (3.7%) | 11 (6.7%) | 1.0 |
| Thirst or frequent urination | 46 (24.1%) | 2 (7.4%) | 44 (26.8%) | 0.029 |
| Weakness, fatigue or malaise | 118 (61.8%) | 16 (59.3%) | 102 (62.2%) | 0.772 |
| Coma Scale § |  |  |  |  |
| 1 | 159 (83.2%) | 21 (77.8%) | 138 (84.1%) | 0.437 |
| 2 | 28 (14.7%) | 5 (18.5%) | 23 (14.0%) |  |
| 3 | 4 (2.1%) | 1 (3.7%) | 3 (1.8%) |  |
| ***Triggers*** | | | | |
| Infectious illness | 68 (35.6%) | 10 (37.0%) | 58 (35.4%) | 0.867 |
| Post-surgery | 8 (4.2%) | 2 (7.4%) | 6 (3.7%) | 0.315 |
| Reduced insulin | 86 (45.0%) | 6 (22.2%) | 80 (48.8%) | 0.010 |
| Alcohol | 2 (1.0%) | 0 (0.0%) | 2 (1.2%) | 1.0 |
| Poor intake | 29 (15.2%) | 5 (18.5%) | 24 (14.6%) | 0.570 |
| Dehydration | 16 (8.4%) | 4 (14.8%) | 12 (7.3%) | 0.251 |
| Other ^†^ | 9 (4.7%) | 5 (18.5%) | 4 (2.4%) | 0.003 |
| Concomitant medication ^‡^ | 6 (3.1%) | 0 (0.0%) | 6 (3.7%) | 0.597 |
| Unknown | 41 (21.5%) | 6 (22.2%) | 35 (21.3%) | 0.918 |

† Other: physical trauma, a recent fall two days earlier resulting in rib fractures, pulmonary congestion due to heart failure following a missed dose of Furosemide, a separate episode of resuscitation upon arrival, ketogenic diet, Addison disease, obstructive stone in the urinary tract, partial bowl obstruction, increased intake of sweets.
‡ Concomitant medication - other than SGLT2 inhibitors, steroids – dose equivalents of over 5 mg prednisone.
§ Coma scale: 1- alert, 2- confused, 3- unresponsive.

**Data are presented as n (%)**

**Abbreviations:** SGLT2i, sodium-glucose cotransporter 2 inhibitor.

## ***Table S2- Multivariate analysis of predictors of DKA severity for the overall population***

| **Multivariable logistic regression including only variables significant in the univariate model** | | | |
| --- | --- | --- | --- |
| **Variable** | **Adjusted OR** | **95% CI** | **p-value** |
| SGLT2i use | 5.483 | 1.807-16.638 | 0.003 |
| eGFR | 1.022 | 1.002-1.042 | 0.029 |
| **Multivariable ANCOVA model including all variables with a significance level < 0.2** | | | |
| **Variable** | **B (Coefficient)** | **95% CI** | **p-value** |
| SGLT2i use | 0.061 | 0.002-0.119 | 0.041 |
| Congestive heart failure | -0.146 | (-0.25) - (-0.043) | 0.006 |
| Creatinine (baseline) | 0.054 | 0.013-0.094 | 0.010 |
| Baseline use of insulin | -0.047 | (-0.101) - 0.007 | 0.091 |
| New onset of DM | -0.05 | (-0.118) - 0.019 | 0.153 |
| Age | -0.001 | (-0.002) - 0.0001 | 0.179 |

Predictors of DKA severity as defined by pH levels.

**Abbreviations**: ANCOVA, analysis of covariance; CI, confidence interval; DKA, diabetic ketoacidosis; DM, diabetes mellitus; eGFR, estimated glomerular filtration rate; OR, odds ratio; SGLT2i, sodium-glucose cotransporter 2 inhibitor.

## ***Table S3- Sensitivity analyses excluding patients with new onset of diabetes***

|  | Overall n = 160 | SGLT2i users n = 27 | SGLT2i non-users n = 133 | p-value |  |
| --- | --- | --- | --- | --- | --- |
| **Baseline Characteristics** | | | | | |
| Age, years | 44.4 ± 20.8 | 65.5 ± 15.7 | 40.2 ± 19.1 | 0.0001 |  |
| Sex |  |  |  |  |  |
| Female | 91 (56.9%) | 10 (37.0%) | 81 (60.9%) | 0.022 |  |
| Male | 69 (43.1%) | 17 (63.0%) | 52 (39.1%) |  |  |
| DM type |  |  |  |  |  |
| Type 1 | 108 (67.5%) | 6 (22.2%) | 102 (76.7%) | 0.0001 |  |
| Type 2 | 48 (30.0%) | 20 (74.1%) | 28 (21.1%) |  |  |
| Other† | 4 (2.5%) | 1 (3.7%) | 3 (2.3%) |  |  |
| Baseline use of insulin | 131 (81.9%) | 13 (48.1%) | 118 (88.7%) | 0.0001 |  |
| Congestive heart failure | 6 (3.8%) | 4 (14.8%) | 2 (1.5%) | 0.008 |  |
| Ischemic heart disease | 16 (10.0%) | 7 (25.9%) | 9 (6.7%) | 0.007 |  |
| **Laboratory Values** | | | | | |
| Nadir pH | 7.14 ± 0.12 | 7.08 ± 0.15 | 7.15 ± 0.11 | 0.019 |  |
| DKA severity by pH levels |  |  |  |  |  |
| Mild (7.25 < pH < 7.3) | 29 (18.1%) | 1 (3.7%) | 28 (21.1%) | 0.011 |  |
| Moderate (7.0 < pH < 7.24) | 108 (67.5%) | 18 (66.7%) | 90 (67.7%) |  |  |
| Severe (pH < 7.0) | 23 (14.4%) | 8 (29.6%) | 15 (11.3%) |  |  |
| pH (first) | 7.15 ± 0.13 | 7.11 ± 0.17 | 7.16 ± 0.12 | 0.119 |  |
| Glucose (first), mg/dL | 499.53 ± 194.42 | 397.59 ± 171.49 | 520.23 ± 192.82 | 0.002 |  |
| Euglycemic DKA (glucose< 250 mg/dL) | 14 (8.8%) | 5 (18.5%) | 9 (6.8%) | 0.063 |  |
| CO2 levels (first), mmol/L | 26.48 ± 7.65 | 26.69 ± 8.92 | 26.44 ± 7.4 | 1.0 |  |
| Anion Gap | 26.27 ± 7.22 | 28.33 ± 8.44 | 25.87 ± 6.92 | 0.112 |  |
| Creatinine (at time of admission), mg/dL | 1.56 ± 0.89 | 1.73 ± 0.71 | 1.52 ± 0.92 | 0.278 |  |
| Baseline creatinine, mg/dL | 0.72 ± 0.48 | 0.85 ± 0.51 | 0.69 ± 0.47 | 0.255 |  |
| eGFR, ml/min/1.73m^2^ | 112.19 ± 29.92 | 91.37 ± 32.75 | 116.42 ± 29.92 | 0.0001 |  |
| eGFR categories, ml/min/1.73m^2^ (baseline) |  |  |  |  |  |
| ≥ 60 | 146 (91.3%) | 21 (77.8%) | 125 (94.0%) | 0.015 |  |
| 60 > eGFR | 14 (8.8%) | 6 (22.2%) | 8 (6.0%) |  |  |
| **Clinical Outcomes** | | | | | |
| Inpatient mortality | 6 (3.8%) | 3 (11.1%) | 3 (2.2%) | 0.061 |  |
| ICU hospitalization | 114 (71.3%) | 17 (63.0%) | 97 (72.9%) | 0.297 |  |
| Mean time in ICU | 2.02 ± 2.71 | 1.52 ± 1.53 | 2.12 ± 2.89 | 0.357 |  |
| Mortality and/or ICU | 118 (73.8%) | 20 (74.1%) | 98 (73.7%) | 0.967 |  |
| Duration of hospitalization, days | 5.9 ± 5.8 | 6.9 ± 7.4 | 5.7 ± 5.5 | 0.500 |  |

† Other – maturity onset diabetes of the young, pancreatitis, other.

Data are presented as mean ± SD or n (%), as appropriate. **Abbreviations**: CO₂, carbon dioxide; DKA, diabetic ketoacidosis; DM, diabetes mellitus; eGFR, estimated glomerular filtration rate; ICU, intensive care unit; SD, standard deviation; SGLT2i, sodium-glucose cotransporter 2 inhibitor.

### ***Table S4- Multivariate analysis of predictors of DKA severity excluding patients with new diabetes***

| Variable | Adjusted OR | 95% CI | p-value |
| --- | --- | --- | --- |
| SGLT2i use | 5.979 | 1.888-18.936 | 0.002 |
| eGFR | 1.023 | 1.002-1.046 | 0.035 |

Predictors of DKA severity as defined by pH levels.

**Abbreviations**: CI, confidence interval; DKA, diabetic ketoacidosis; eGFR, estimated glomerular filtration rate; OR, odds ratio; SGLT2i, sodium-glucose cotransporter 2 inhibitor.

***Subgroup analysis by diabetes type***

### ***Table S5- Subgroup analysis of T1DM patients***

|  | **Overall n = 132** | **SGLT2i users n = 6** | **SGLT2i non-users n = 126** | **p-value** |  |
| --- | --- | --- | --- | --- | --- |
| **Baseline Characteristics** | | | | | |
| Age, years | 35.1 ± 17.1 | 60.2 ± 11.6 | 33.9 ± 16.4 | 0.001 |  |
| Sex |  |  |  |  |  |
| Female | 77 (58.3%) | 3 (50.0%) | 74 (58.7%) | 0.693 |  |
| Male | 55 (41.7%) | 3 (50.0%) | 52 (41.3%) |  |  |
| Baseline use of insulin | 103 (78.0%) | 5 (83.3%) | 98 (77.8%) | 1.000 |  |
| Congestive heart failure | 1 (0.8%) | 1 (16.7%) | 0 (0%) | 0.045 |  |
| Ischemic heart disease | 3 (2.3%) | 0 (0%) | 3 (2.4%) | 1.000 |  |
| **Laboratory Values** | | | | | |
| Nadir pH | 7.14±0.13 | 7.08±0.14 | 7.14±0.12 | 0.239 |  |
| DKA severity by pH levels |  |  |  |  |  |
| Mild (7.25 < pH < 7.3) | 33 (25.0%) | 0 (0%) | 33 (26.2%) | 0.243 |  |
| Moderate (7.0 < pH < 7.24) | 78 (59.1%) | 4 (66.7%) | 74 (58.7%) |  |  |
| Severe (pH < 7.0) | 21 (15.9%) | 2 (33.3%) | 19 (15.1%) |  |  |
| Glucose (first), mg/dL | 492.92±170.6 | 457.33±140.9 | 494.61±172.15 | 0.603 |  |
| Euglycemic DKA (glucose< 250 mg/dL) | 10 (7.6%) | 1 (16.7%) | 9 (7.1%) | 0.383 |  |
| eGFR categories, ml/min/1.73m^2^ (baseline) |  |  |  |  |  |
| ≥ 60 | 126 (95.5%) | 5 (83.3%) | 121 (96.0%) | 0.248 |  |
| 60 > eGFR | 6 (4.5%) | 1 (16.7%) | 5 (4.0%) |  |  |
| **Clinical Outcomes** | | | | | |
| Inpatient mortality | 1 (0.8%) | 0 (0%) | 1 (0.8%) | 1.000 |  |
| ICU hospitalization | 100 (75.8%) | 6 (100%) | 94 (74.6%) | 0.335 |  |
| Mortality and/or ICU | 100 (75.8%) | 6 (100%) | 94 (74.6%) | 0.335 |  |
| Duration of hospitalization, days | 4.9 ± 5.3 | 8.0±10.8 | 4.7±5.0 | 0.495 |  |

Data are presented as mean ± SD or n (%), as appropriate.

**Abbreviations**: DKA, diabetic ketoacidosis; eGFR, estimated glomerular filtration rate; ICU, intensive care unit; SD, standard deviation; SGLT2i, sodium-glucose cotransporter 2 inhibitor; T1DM, type 1 diabetes mellitus.

### ***Table S6- Subgroup analysis of T2DM patients***

|  | Overall n = 53 | SGLT2i users n = 20 | SGLT2i non-users n = 33 | **p-value** |
| --- | --- | --- | --- | --- |
| **Baseline Characteristics** | | | | |
| Age, years | 61.4 ±17.3 | 67.5±16.8 | 57.7± 16.7 | 0.046 |
| Sex |  |  |  |  |
| Female | 23 (43.4%) | 7 (35.0%) | 16 (48.5%) | 0.337 |
| Male | 30 (56.6%) | 13 (65.0%) | 17 (51.5%) |  |
| Baseline use of insulin | 25 (47.2%) | 7 (35.0%) | 18 (54.5%) | 0.167 |
| Congestive heart failure | 5 (9.4%) | 3 (15.0%) | 2 (6.1%) | 0.354 |
| Ischemic heart disease | 13 (24.5%) | 7 (35.0%) | 6 (18.2%) | 0.200 |
| **Laboratory Values** | | | | |
| Nadir pH | 7.12±0.13 | 7.07±0.16 | 7.15±0.1 | 0.036 |
| DKA severity by pH levels |  |  |  |  |
| Mild (7.25 < pH < 7.3) | 6 (11.3%) | 1 (5.0%) | 5 (15.2%) | 0.013 |
| Moderate (7.0 < pH < 7.24) | 40 (75.5%) | 13 (65.0%) | 27 (81.8%) |  |
| Severe (pH < 7.0) | 7 (13.2%) | 6 (30.0%) | 1 (3.0%) |  |
| Glucose (first), mg/dL | 494.74±214.76 | 368.75±174.63 | 571.09±202.258 | <0.001 |
| Euglycemic DKA (glucose< 250 mg/dL) | 4 (7.5%) | 4 (20.0%) | 0 (0%) | 0.017 |
| eGFR categories, ml/min/1.73m^2^ (baseline) |  |  |  |  |
| ≥ 60 | 44 (83.0%) | 15 (75.0%) | 29 (87.9%) | 0.272 |
| 60 > eGFR | 9 (17.0%) | 5 (25.0%) | 4 (12.1%) |  |
| **Clinical Outcomes** | | | | |
| Inpatient mortality | 5 (9.4%) | 3 (15.0%) | 2 (6.1%) | 0.354 |
| ICU hospitalization | 31 (58.5%) | 10 (50.0%) | 21 (63.6%) | 0.329 |
| Mortality and/or ICU | 35 (66.0%) | 13 (65.0%) | 22 (66.7%) | 0.901 |
| Duration of hospitalization, days | 8.3±7.5 | 5.7±5.1 | 9.9±8.3 | 0.051 |

### Data are presented as mean ± SD or n (%), as appropriate.

### **Abbreviations**: DKA, diabetic ketoacidosis; eGFR, estimated glomerular filtration rate; ICU, intensive care unit; SD, standard deviation; SGLT2i, sodium-glucose cotransporter 2 inhibitor; T2DM, type 2 diabetes mellitus.
